# Impact of Obesity on Systemic Treatment Outcomes in Metastatic Urological Malignancies

**DOI:** 10.1002/osp4.70164

**Published:** 2026-06-05

**Authors:** A. Handke, M. Silberg, C. Orf, C. Loeffelholz, P. Bach, S. Berg, F. Roghmann, K. H. Tully

**Affiliations:** ^1^ Department of Urology and Neurourology Marien Hospital Herne, Ruhr‐University Bochum Bochum Germany

**Keywords:** bladder cancer, obesity, overall survival, prostate cancer, renal cell cancer, systemic therapies

## Abstract

**Introduction:**

Obesity increasingly influences treatment outcomes in urological malignancies (metastatic bladder cancer (BC), renal cell cancer (RCC), and prostate cancer (PC)). This study evaluated the impact of obesity on survival in patients with major metastatic urological cancers receiving systemic therapy.

**Materials and Methods:**

Patients with metastatic BC, RCC, and PC treated at a high‐volume center from 2017 to 2025 were retrospectively analyzed. In total, 387 patients were included (BC: *n* = 166; RCC: *n* = 63; PC: *n* = 158) and stratified by the body mass index [BMI]. Overall survival was assessed using restricted mean survival time (RMST) at predefined landmarks (24 months for BC and RCC; 36 months for PC).

**Results:**

Overall, 23.5% of the patients had obesity, with the highest prevalence in RCC (44.4%). In metastatic BC, obesity was not associated with differences in RMST at 24 months. In RCC, patients with obesity showed a numerical improvement in RMST and a significantly longer duration of systemic therapy. In contrast, patients with obesity with metastatic PC demonstrated significantly shorter RMST at 36 months than patients without obesity.

**Conclusion:**

The prognostic impact of obesity differs across metastatic urological malignancies. While the present findings support an obesity paradox in RCC, obesity was associated with worse survival in metastatic PC but showed no impact on survival in BC. These results highlight the need for tumor‐specific biological interpretation of BMI‐related outcomes.

## 1 Introduction

Urological malignancies, including bladder cancer (BC), renal cell carcinoma (RCC), and prostate cancer (PC), pose a significant global health burden. Obesity is recognized not only as a risk factor for cancer development but also as a factor that influences treatment outcomes. Its interaction with systemic therapies has garnered considerable interest, showing complex and sometimes paradoxical relationships.

Obesity creates a tumor‐promoting environment through multiple interconnected pathways, including disrupted adipokine levels (e.g., elevated leptin and reduced adiponectin), insulin resistance, and persistent low‐grade inflammation [1, 2, 3, 4]. These alterations collectively affect cancer cell proliferation, angiogenesis, and immune system function [1, 5]. In particular, imbalances in adipokines and the release of pro‐inflammatory cytokines contribute to a microenvironment that favors tumor growth, new blood vessel formation, and immune escape [1, 3, 6, 7]. Elevated insulin levels and insulin resistance further stimulate pro‐growth signaling pathways and angiogenesis, while chronic inflammatory states attract immune cells that support tumor progression [2, 4, 5, 8, 9]. Additionally, these metabolic and molecular changes may influence how anticancer drugs are absorbed, distributed, metabolized, and eliminated, potentially impacting treatment efficacy and toxicity in obese patients [10, 11, 12, 13, 14]. Therefore, further research is needed to clarify whether obesity‐associated biological differences influence treatment tolerance, toxicity profiles, or monitoring requirements during systemic therapy.

In BC, the association between obesity and systemic treatment outcomes is poorly defined. Meta‐analyses indicate a modest increase in risk with higher BMI, yet data on its impact on therapy response and survival remain limited [15, 16, 17].

In RCC, obesity presents a paradox: while higher BMI increases RCC risk, overweight and patients with obesity often show improved overall and progression‐free survival under systemic therapy, including ICIs and targeted treatments [18, 19]. These findings suggest that BMI may function as an independent prognostic factor, though mechanisms remain incompletely understood.

In PC, obesity is linked to more aggressive disease and higher cancer‐specific mortality, likely mediated by inflammatory processes [20, 21]. Large meta‐analyses indicate a dose‐dependent increase in mortality with higher BMI, yet evidence on the impact of obesity during systemic therapy in metastatic PC is scarce [22].

Despite growing interest in the interaction between obesity and systemic anticancer therapy, existing evidence across urological malignancies remains fragmented and often entity‐specific. Most available studies have focused on single tumor entities, selected treatment settings, or post hoc analyses of clinical trial populations, limiting direct comparability across diseases and therapeutic contexts. In particular, comparative real‐world data evaluating the prognostic impact of obesity across metastatic BC, RCC, and PC within a unified clinical framework are scarce. Moreover, contemporary treatment strategies, including immune checkpoint inhibitor‐based combinations and intensified systemic approaches, may modify previously reported BMI‐associated survival patterns. Therefore, the present study aimed to evaluate the association between obesity and systemic treatment outcomes across major metastatic urological malignancies treated at a high‐volume tertiary center using a consistent analytical approach based on restricted mean survival time and treatment duration.

## 2 Materials and Methods

### 2.1 Study Design

This retrospective observational study was conducted at a high‐volume uro‐oncology center. Patients who received systemic therapy for metastatic BC, RCC, or PC between January 2017 and December 2025 were identified through institutional electronic medical records. Ethical approval for the study was obtained from the institutional review board (Reg.‐No.: 16‐5913 and Reg.‐No.: 4047‐11), and all procedures were conducted in accordance with the Declaration of Helsinki.

### 2.2 Patient Selection

Eligible patients were adults (≥ 18 years) with histologically confirmed metastatic BC, RCC, or PC who received systemic therapy within the study period and had documented baseline BMI and complete therapeutic data. Exclusion criteria included missing BMI measurements or incomplete survival information. The final cohort consisted of 387 patients: 166 BC, 63 RCC, and 158 PC cases.

### 2.3 Data Collection

Clinical, pathological, and treatment‐related data were retrieved from institutional databases. For BC and RCC, variables included age, sex, ECOG performance status, histologic subtype, TNM classification, metastatic sites (lung, liver, lymph nodes, bone, and others), treatment modality (chemotherapy, immune checkpoint inhibitors, targeted therapies), treatment line, and treatment duration. For PC, additional variables included comorbidities, baseline prostate‐specific antigen (PSA) levels, C‐reactive protein (CRP) levels, and laboratory data. BMI was calculated at the initiation of systemic therapy and categorized according to WHO definitions: patients with normal weight (BMI < 30 kg/m^2^) and obesity (BMI ≥ 30 kg/m^2^).

### 2.4 Outcome Measures

The primary endpoint was overall survival (OS), defined as the time from initiation of systemic therapy to death from any cause or last known follow‐up. OS was assessed using restricted mean survival time (RMST) at predefined landmarks: 24 months for BC and RCC, and 36 months for PC. Secondary endpoints included progression‐free survival (where available), total duration of systemic therapy, distribution of metastatic sites by BMI, and, for PC, differences in baseline characteristics, PSA levels, and CRP levels between BMI groups. Follow‐up duration was calculated from the start of systemic therapy to the last contact or death.

### 2.5 Statistical Analysis

Medians and interquartile ranges (IQRs) were generated for continuous variables, and frequencies and proportions for categorical variables. Then, the Mann–Whitney U‐test and the chi‐square test were employed to examine differences in continuous and categorical variables, respectively. The primary analyses were based on restricted mean survival time (RMST). Overall survival was assessed using restricted mean survival time (RMST) at predefined time points. For all time‐dependent analyses, time zero was defined as the time of treatment initiation for metastatic disease. Finally, separate Mann‐Whitney *U* tests were used to examine differences in median therapy duration among patients receiving systemic therapy. All analyses were conducted using Stata (Version Stata/SE 15.1, Stata Corp LLC, TX, USA). A two‐sided *p*‐value < 0.05 was considered statistically significant.

## 3 Results

### 3.1 Patient Characteristics

A total of 387 patients were included in the analysis: 166 with metastatic bladder cancer (BC), 63 with renal cell carcinoma (RCC), and 158 with prostate cancer (PC). The overall prevalence of obesity (BMI ≥ 30 kg/m^2^) was 23%–24%, with the highest proportion in RCC (44.4%) compared to BC (23%; *p* = 0.002) and PC (22.8%). Median age at treatment initiation was 67 years (IQR 61–75 years) for BC, 65 years (IQR 59–72 years) for RCC, and 66 years (IQR 60–73 years) for PC, with no significant differences between patients with obesity and non‐obesity within each cohort (BC: *p* = 0.199, RCC: *p* = 0.128, PC: *p* = 0.580).

In BC, patients presented less frequently with bone metastases at the baseline (13.8% vs. 33%; *p* = 0.045), whereas rates of liver (19.6% vs.14.8%; *p* = 0.478), lung (18.6% vs. 13.8%; *p* = 0.553), and lymph‐node metastases (70.1% vs. 72.4%; *p* = 0.811) did not differ significantly. In RCC and PC, the distribution of metastatic sites showed no statistically significant differences between patients with non‐obesity and obesity (Table 1).

**TABLE 1:** Baseline patient and therapy characteristics.

a) Bladder cancer		Patients with non‐obesity *n* = 97	Patients with obesity *n* = 29	*p* value
Median age at diagnosis, years (IQR)		67 (61–75)	65 (58–71)	0.199
Median BMI^a^ at diagnosis, kg/m^2^ (IQR)		24.91 (23.31–27.34)	32.32 (31.02–34.48)	< 0.001
Metastatic setting, *n* (%)	Synchronous	28 (28.87)	6 (20.69)	0.384
	Metachronous	69 (71.13)	23 (79.31)	
First line therapy, *n* (%)	Gemcitabin/cisplatin	63 (64.95)	18 (62.07)	0.894
	Gemcitabin/carboplatin	32 (32.99)	10 (34.48)	
	Pembrolizumab	2 (2.06)	1 (3.45)	
Median baseline C‐reactive protein, mg/dl (IQR)		2 (0.6–6.6)	1 (0.4–4.6)	0.196
Lymph node positive disease, *n* (%)	Yes	68 (70.10)	21 (72.41)	0.811
	No	29 (29.90)	8 (27.59)	
Bone metastases, *n* (%)	Yes	32 (32.99)	4 (13.79)	0.045
	No	65 (67.01)	25 (86.21)	
Lung metastases, *n* (%)	Yes	18 (18.55)	4 (13.79)	0.553
	No	79 (81.45)	25 (86.21)	
Liver metastases, *n* (%)	Yes	19 (19.59)	4 (13.79)	0.478
	No	78 (80.41)	25 (86.21)	

**b) Renal cell carcinoma**		***n* = 35**	***n* = 28**	
Median age at diagnosis, years (IQR)		66 (59–75)	64 (58–68)	0.138
Median BMI^a^ at diagnosis, kg/m^2^ (IQR)		23.84 (21.45–26.49)	33.69 (31.31–35.56)	< 0.001
Metastatic setting, *n* (%)	Synchronous	4 (11.43)	7 (25.0)	0.159
	Metachronous	31 (88.57)	21 (75.0)	
First line therapy, *n* (%)	IO/TKI	24 (68.57)	18 (64.28)	0.811
	IO/IO	2 (5.71)	1 (3.57)	
	TKI‐monotherapy	9 (25.71)	9 (32.14)	
Median baseline C‐reactive protein, mg/dl (IQR)		1.3 (0.3–8.1)	2 (0.3–5.6)	0.861
Lymph node positive disease, *n* (%)	Yes	19 (54.29)	13 (46.43)	0.473
	No	16 (45.71)	15 (63.57)	
Bone metastases, *n* (%)	Yes	11 (31.43)	13 (46.43)	0.223
	No	24 (68.57)	15 (53.57)	
Lung metastases, *n* (%)	Yes	20 (57.14)	11 (39.29)	0.159
	No	15 (42.86)	17 (60.71)	
Liver metastases, *n* (%)	Yes	8 (22.86)	2 (7.14)	0.090
	No	27 (77.14)	26 (92.86)	

**c) Prostate cancer**		***n* = 122**	***n* = 36**	
Median age at diagnosis, years (IQR)		66 (61–73)	66 (58–73)	0.580
Median BMI^a^ at diagnosis, kg/m^2^ (IQR)		26.35 (24.49–28.34)	33.58 (32.61–36.64)	< 0.001
Median PSA^b^ at diagnosis, ng/mL (IQR)		77.5 (18–544)	50 (8.8–188)	0.447
Metastatic setting, *n* (%)	Synchronous	46 (37.71)	19 (52.77)	0.106
	Metachronous	76 (62.29)	17 (47.23)	
First line therapy, *n* (%)	ADT only	36 (29.51)	16 (44.44)	0.212
	ARPI^c^	23 (18.85)	7 (19.44)	
	Docetaxel	37 (30.3)	10 (27.8)	
	Docetaxel + ARPI	26 (21.31)	3 (8.33)	
Median baseline PSA at diagnosis of metastatic disease, mg/dl (IQR)		77.5 (18–544)	50 (8.8–188)	0.447
Median baseline C‐reactive protein, mg/dl (IQR)		0.3 (0.1–0.95)	0.3 (0.2–1.2)	0.580
Lymph node positive disease, *n* (%)	Yes	89 (72.95)	24 (66.67)	0.463
	No	33 (27.05)	12 (33.33)	
Bone metastases, *n* (%)	Yes	101 (82.79)	30 (83.33)	0.182
	No	21 (17.21)	6 (16.67)	
Lung metastases, *n* (%)	Yes	10 (8.19)	4 (11.11)	0.589
	No	112 (91.81)	32 (88.89)	
Liver metastases, *n* (%)	Yes	117 (95.90)	34 (94.44)	0.709
	No	5 (4.10)	2 (5.55)	

^a^ BMI = Body mass index.

^b^ PSA = Prostate‐specific antigen.

^c^ ARPI = Androgen receptor pathway inhibitor.

The median follow‐up duration was 8.5 months (IQR 2–21) for bladder cancer, 11.5 months (IQR 4–26) for renal cell carcinoma, and 27 months (IQR 11–44) for prostate cancer.

### 3.2 Overall Survival and RMST Analysis

#### 3.2.1 Bladder Cancer

**FIGURE 1:**
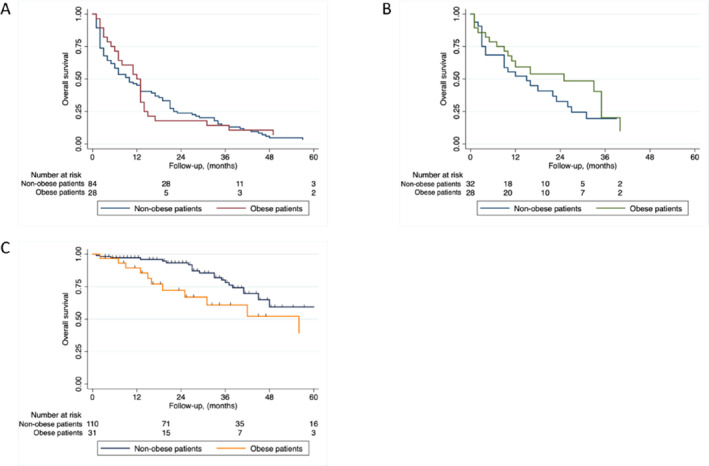
Overall survival of (A) bladder cancer (B) renal cell carcinoma (C) prostate cancer.

In metastatic BC, no difference in overall survival (OS) was observed between the BMI groups. The RMST at 24 months was 11.7 months for patients with obesity and 11.9 months for patients with non‐obesity (*p* = 0.907). Median OS was 12 months (IQR 5–14 months) in patients with obesity versus 10 months (IQR 2–22 months) in patients with non‐obesity, and Kaplan‐Meier curves showed no clinically relevant separation (log‐rank *p* = 0.693) (Figure 1A). (Table 2)

**TABLE 2:** Survival analysis at the 12‐, 24‐, and 36‐month analysis using restricted mean survival time.

Landmark	Patients with non‐obesity, months (95% CI)	Patients with obesity, months (95% CI)	*p* value
a) Bladder cancer			
12‐month	7.7 (6.7–8.7)	8.9 (7.3–10.6)	0.197
24‐month	11.9 (9.9–13.9)	11.7 (8.7–14.7)	0.907
36‐month	14.3 (11.5–17.1)	13.6 (9.1–18.2)	0.803
b) Renal cell carcinoma			
12‐month	8.7 (7.0–10.4)	9.5 (8.2–10.7)	0.447
24‐month	13.9 (10.5–17.5)	16.2 (13.0–19.4)	0.361
36‐month	16.9 (11.9–21.9)	21.6 (16.1–27.2)	0.213
c) Prostate cancer			
12‐month	11.7 (11.5–11.9)	11.4 (10.9–11.9)	0.208
24‐month	23.2 (22.5–23.8)	20.7 (18.6–22.9)	0.034
36‐month	33.6 (32.2–34.9)	28.5 (24.3–32.8)	0.027

#### 3.2.2 Renal Cell Carcinoma

RCC patients with obesity exhibited a trend toward improved survival. RMST at 24 months was 16.2 months in patients with obesity versus 13.9 months in patients with non‐obesity (*p* = 0.361). Similarly, median OS was 25 months (IQR 7–35 months) for patients with obesity versus 15 months (IQR 3–27 months) for patients with non‐obesity (*p* = 0.3543). Kaplan‐Meier curves indicated a consistent numerical survival advantage in patients with obesity, though differences were not statistically significant (Figure 1B). (Table 2)

#### 3.2.3 Prostate Cancer

At the 24‐ and 36‐month landmarks, PC patients with obesity had shorter RMSTs than patients with non‐obesity (24 months: 20.7 vs. 23.2 months, *p* = 0.034; 36 months: 28.5 vs. 33.6 months, *p* = 0.027) (Figure 1C). Baseline characteristics, PSA levels, CRP levels, and metastatic distribution were similar between the groups. No significant differences in response to specific systemic therapies were observed, although subgroup sample sizes were limited (Table 2).

### 3.3 Therapy Duration

**FIGURE 2:**
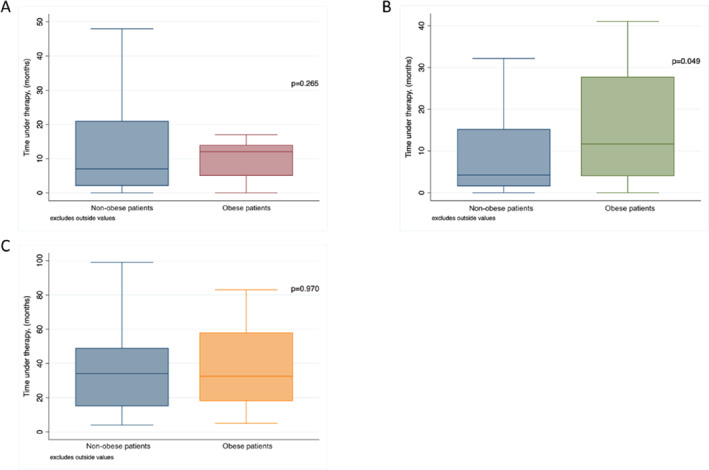
Median time under therapy of (A) bladder cancer (B) renal cell carcinoma (C) prostate cancer.

In BC, the therapy duration was 12 months (IQR 5–24 months) for patients with obesity versus 7 months (IQR 2–21 months) for patients with non‐obesity (*p* = 0.265), with no significant difference (Figure 2A). In RCC, patients with obesity received systemic therapy for a median of 11.7 months (IQR 3.9–27.8 months) versus 4.2 months (IQR 1.5–15.2 months) in patients with non‐obesity (*p* = 0.049) (Figure 2B). Therapy duration in PC was not significantly different between BMI groups (Figure 2B).

## 4 Discussion

In this retrospective cohort of patients with metastatic urological malignancies treated with systemic therapy, the prognostic impact of obesity differed substantially between tumor entities, underscoring the biological heterogeneity of BC, RCC, and PC.

In patients with metastatic bladder cancer treated with systemic therapy, obesity was not associated with overall survival, restricted mean survival time, or treatment duration. This observation is consistent with the heterogeneous and context‐dependent evidence in bladder cancer [15, 16, 17]. It suggests that the prognostic relevance of obesity is strongly influenced by the disease stage and treatment modality.

In surgically treated muscle‐invasive BC, several large series have reported worse oncologic outcomes in patients with obesity following radical cystectomy, including higher recurrence rates and cancer‐specific mortality after adjustment for pathological features [23]. However, other cohorts have observed improved short‐term overall survival in overweight patients and those with obesity without corresponding differences in cancer‐specific survival, with this advantage diminishing over longer follow‐up, suggesting a time‐limited obesity paradox potentially driven by competing non‐cancer mortality [16]. Additional multicenter data indicate that obesity increases perioperative morbidity without clearly affecting long‐term survival, implying that BMI‐related effects in localized disease may primarily reflect surgical risk rather than durable oncologic control [24].

In contrast, evidence from metastatic urothelial carcinoma treated with immune checkpoint inhibitors suggests that a higher BMI is not detrimental and may even be associated with improved overall survival. Across contemporary cohorts, underweight patients consistently exhibit the poorest outcomes, whereas normal‐weight, overweight, and patients with obesity demonstrate comparable or superior survival [25, 26]. These findings imply that BMI‐related associations in metastatic disease may be driven largely by the adverse prognostic impact of low BMI rather than by a direct protective effect of obesity.

Against this backdrop, the absence of a survival difference between patients with obesity and non‐obesity in the metastatic BC cohort appears biologically plausible. Heterogeneity in BMI definitions, endpoints, and treatment context likely contributes to divergent findings across studies. Moreover, BMI‐associated survival differences in immunotherapy‐treated populations are typically more pronounced for overall survival than for progression‐free survival, suggesting an influence of treatment tolerance, performance status, or competing risks rather than direct tumor control. Although patients with obesity in this cohort exhibited a lower frequency of bone metastases, this did not translate into improved survival and may therefore represent a confounded association.

Overall, the present findings indicate that obesity is not a strong independent prognostic factor in metastatic bladder cancer treated with systemic therapy, reinforcing the importance of disease stage and treatment context when interpreting BMI‐related outcomes.

Obesity is a well‐established causal risk factor for renal cell carcinoma development. Yet, in a metastatic setting, an inverse association between BMI and survival under systemic therapy has been consistently reported, giving rise to an obesity paradox. Across large international cohorts and meta‐analyses, higher BMI in metastatic RCC treated with VEGF‐targeted therapies is associated with improved overall and progression‐free survival, with effects largely consistent across treatment lines and agents [27]. Conflicting results from smaller studies using alternative body composition measures likely reflect methodological heterogeneity rather than true biological inconsistency. This body of evidence provides context for the observation of longer treatment duration and a numerical survival advantage in RCC patients with obesity, suggesting enhanced treatment tolerance or prolonged disease control.

Data from the immunotherapy era are more heterogeneous but generally support the same direction of effect, particularly for ICI‐TKI combinations, *w*. In contrast, progression‐free survival benefits are less consistent and may be absent with ICI monotherapy [28]. Underweight patients consistently exhibit the poorest outcomes, indicating that adverse prognosis is driven primarily by low BMI rather than obesity itself. The survival trend observed in patients with obesity in this cohort treated with contemporary ICI‐based regimens is therefore concordant with current real‐world data.

Biologically, obesity‐associated inflammation, altered lipid metabolism, and immune modulation may increase tumor dependence on angiogenic and immune checkpoint pathways, potentially enhancing responsiveness to VEGF‐targeted therapies and selected immunotherapy regimens [27, 29]. However, mechanistic evidence is not uniform. Translational data suggesting impaired responses to PD‐1 monotherapy in patients with obesity highlight that obesity‐related effects may be regimen‐specific rather than universal [30]. Importantly, these studies primarily evaluated PD‐1 monotherapy and implicated cell‐mediated immunosuppressive mechanisms, limiting direct comparability to contemporary cohorts dominated by combination ICI‐TKI regimens.

Overall, these findings support the concept of an obesity paradox in metastatic RCC, as reflected by prolonged treatment persistence and numerically improved survival in patients with obesity, while underscoring the importance of treatment context and biological heterogeneity.

In metastatic PC, a growing body of evidence suggests that higher BMI is associated with improved overall survival once systemic therapy is initiated, particularly in the castration‐resistant setting, despite obesity being linked to worse prognosis across the overall prostate cancer disease course. This apparent contradiction can largely be explained by disease state and timing.

Although obesity is associated with increased PC‐specific mortality and faster progression in localized and hormone‐sensitive disease, multiple large metastatic cohorts consistently demonstrate a survival advantage for overweight patients once systemic therapy is started, giving rise to a context‐dependent obesity paradox [31, 32, 33, 34]. A large meta‐analysis encompassing more than 18,000 men with metastatic disease confirmed this pattern, with the most potent effects observed in mCRPC and a dose‐response relationship across BMI categories [31].

This association is particularly robust in mCRPC treated with docetaxel‐based chemotherapy, where pooled analyses of large randomized trial cohorts showed significantly longer overall survival and lower cancer‐specific mortality in men with obesity after adjustment for tumor burden, PSA, and performance status [32]. Notably, the survival advantage appears therapy‐specific, being most consistently observed with docetaxel, androgen receptor pathway inhibitors, and PSMA‐directed radioligand therapy. In contrast, evidence is weaker or more heterogeneous for other treatment modalities [32, 33, 34, 35]. In metastatic hormone‐sensitive disease, patients with high obesity seem to derive less incremental benefit from treatment intensification beyond ADT, suggesting that extreme adiposity may attenuate the relative gains of combination therapy in earlier metastatic settings [36].

Across studies, differences in reported outcomes are also strongly influenced by the choice of endpoints and covariate adjustment. PFS advantages are often modest or absent, as illustrated by post hoc analyses of docetaxel trials, whereas overall survival benefits persist in large, well‐adjusted datasets [35, 37]. In smaller or single‐center series, the prognostic effect of BMI may disappear once laboratory markers such as hemoglobin or LDH are included, indicating that BMI partly captures underlying disease burden or physiologic reserve rather than acting as an independent causal factor [37].

A key source of heterogeneity is confounding by weight loss and frailty. Low BMI at metastatic presentation frequently reflects cancer‐related cachexia, systemic inflammation, or poor performance status, which are themselves powerful predictors of inferior survival [34, 37]. Accordingly, several cohorts identify underweight patients as the group with the worst outcomes, supporting the interpretation that preserved or higher BMI functions as a marker of metabolic and functional reserve during systemic treatment [33, 34]. This concept is consistent with the current findings, where patients with obesity did not differ in baseline disease characteristics but experienced shorter restricted mean survival times under systemic therapy, suggesting additional unmeasured metabolic or hormonal modifiers.

From a biological standpoint, adipose‐driven inflammation, altered sex‐steroid signaling, activation of the insulin/IGF axis, and changes in drug pharmacokinetics and immune modulation have been proposed as mechanisms that may simultaneously promote prostate cancer development while enhancing the tolerance or efficacy of systemic therapies once treatment has begun [38, 39]. These mechanisms provide a plausible framework for why obesity may worsen long‐term disease risk but confer a survival advantage in the metastatic, treatment‐exposed population.

Taken together, these data indicate that the obesity paradox in metastatic prostate cancer is highly context‐dependent, shaped by disease stage, therapy type, endpoint selection, and residual confounding by weight loss and frailty. The findings of this study are concordant with large contemporary cohorts showing improved overall survival in higher‐BMI patients receiving systemic therapy, while underscoring that BMI should be interpreted as a composite marker of host reserve and treatment interaction rather than a direct biological protector.

Several limitations should be acknowledged. First, the retrospective single‐center design introduces potential selection bias and residual confounding. Second, BMI was the sole measure of obesity, which does not capture body composition, sarcopenia, or metabolic health factors that may be more prognostically relevant. Third, systemic treatment regimens were heterogeneous and reflected the rapidly evolving therapeutic landscape between 2017 and 2025, including the increasing use of immune checkpoint inhibitor‐based combinations and intensified systemic approaches, which may limit comparability across treatment eras. Fourth, subgroup sample sizes, particularly in the RCC cohort, were limited relative to the study period and reduced statistical power for entity‐specific subgroup analyses. Finally, detailed metabolic, hormonal, and immunological biomarkers were unavailable, precluding mechanistic insights into entity‐specific effects of obesity.

Taken together, the present findings highlight that the prognostic relevance of obesity in metastatic urological malignancies is highly entity‐specific. While obesity may confer a survival advantage in immunologically driven tumors such as RCC, it appears neutral in BC and detrimental in hormonally and metabolically sensitive tumors such as PC. These divergent effects underscore the need for tumor‐specific biological investigations and caution against generalizing about obesity's role across urological cancers.

## 5 Conclusion

In this retrospective cohort of metastatic urological malignancies, the prognostic impact of obesity was highly tumor‐specific. Obesity was not independently associated with survival in metastatic bladder cancer. In contrast, an obesity paradox was evident in renal cell carcinoma, characterized by a longer treatment duration and numerically improved survival under systemic therapy. In contrast, obesity was associated with inferior survival outcomes in metastatic prostate cancer, underscoring fundamental differences in tumor biology and host‐tumor interactions across entities. Collectively, these findings highlight that BMI should not be interpreted as a universal prognostic marker in metastatic urological cancers, but rather as a context‐dependent surrogate reflecting entity‐specific biology, treatment modality, and host reserve. Prospective studies incorporating refined body‐composition measures and metabolic biomarkers are warranted to better understand the mechanisms underlying these divergent effects.

## Author Contributions

**A. Handke:** data collection, writing – original draft, formal analysis. **M. Silberg:** data collection, writing – review and editing. **C. Orf:** writing – review and editing. **C. Loeffelholz:** data collection. **P. Bach:** writing – review and editing. **S. Berg:** writing – review and editing. **F. Roghmann:** writing – review and editing. **K. H. Tully:** data analysis, writing – review and editing.

## Funding

The authors have nothing to report.

## Conflicts of Interest

The authors declare no conflicts of interest.

## Data Availability

The data that support the findings of this study are available on request from the corresponding author. The data are not publicly available due to privacy or ethical restrictions.
